# Identification of maturity-onset diabetes of the young through targeted next-generation sequencing in Thai patients with atypical diabetes in real-world practice

**DOI:** 10.3389/fendo.2026.1717121

**Published:** 2026-01-29

**Authors:** Yotsapon Thewjitcharoen, Waralee Chatchomchuan, Ekgaluck Wanothayaroj, Soontaree Nakasatien, Cadmon K. P. Lim, Thep Himathongkam, Juliana C. N. Chan

**Affiliations:** 1 THEPTARIN Diabetes, Thyroid and Endocrine Center, Vimut-Theptarin Hospital, Bangkok, Thailand; 2 Department of Medicine and Therapeutics, The Chinese University of Hong Kong, Prince of Wales Hospital, Hong Kong, Hong Kong SAR, China; 3 Hong Kong Institute of Diabetes and Obesity, The Chinese University of Hong Kong, Prince of Wales Hospital, Hong Kong, Hong Kong SAR, China; 4 Asia Diabetes Foundation, Hong Kong, Hong Kong SAR, China

**Keywords:** atypical diabetes, identification, MODY, monogenic diabetes, Thai

## Abstract

**Introduction:**

Maturity-onset diabetes of the young (MODY) is often misdiagnosed as either autoimmune type 1 diabetes (T1D) or polygenic type 2 diabetes (T2D), resulting in missed diagnosis and inappropriate treatment. Differentiating MODY from T2D is challenging in Asians with low body mass index (BMI) and strong family history. The clinical impact of genetic testing in a real-world case series of Thai patients with atypical diabetes is not well defined. In this study, we aim to evaluate the diagnostic yield and clinical implications of targeted gene panel testing at a specialized diabetes outpatient clinic in Bangkok.

**Materials and methods:**

We performed next-generation sequencing analysis of 33 monogenic diabetes genes in Thai patients recruited in 2019–2025 who had atypical features of diabetes including age-at-diagnosis≤ 40 years, BMI ≤25 kg/m^2^, random plasma C-peptide levels ≥ 0.1 ng/mL after at least three years of clinically-diagnosed T1D, syndromic features such as organ abnormalities or non-classical T1D or T2D presentations.

**Results:**

Of the 33 probands with atypical diabetes (age-at-diagnosis 34.4 ± 14.4 years, BMI 23.7 ± 3.3 kg/m^2^, insulin-treated 39.3%), genetic testing identified a pathogenic or likely pathogenic variant in 4 (12.1%) probands. Variants in *GCK* were the most frequent (n=2, 50.0%), followed by *HNF1A* (n=1, 25.0%), and *HNF1B* (n=1, 25.0%). Genetic diagnoses led to targeted therapies and identification of MODY cases among family members. The latter often have concomitant obesity-driven insulin resistance contributing to hyperglycemia.

**Conclusion:**

Genetic testing for monogenic diabetes in a real-world setting identified disease-causing variant in 12.1% of young Thai patients with atypical diabetes. Despite this low yield, accurate genetic diagnoses improved clinical management in both probands and family members. These findings underscore the potential contribution of a strong polygenic background or yet unidentified MODY-X genes among Thai patients. Establishing a register of family-based cohorts documenting the molecular diagnosis of atypical diabetes will advance diagnosis and treatment.

## Introduction

Maturity-onset diabetes of the young (MODY) is the most common form of monogenic diabetes. This is a clinically and genetically heterogeneous disease with both familial and sporadic manifestation (1–3). To date, over 14 genes have been identified as the cause of different MODY subtypes. A genetic diagnosis in some MODY subtypes can influence clinical management with implications on prognosis, genetic counseling and cascade screening of other family members. Identification of individuals with MODY exemplifies the principles of precision medicine in diabetes care, allowing for personalized treatment strategies and optimized medication selection (4). Despite these benefits, MODY is frequently misdiagnosed as autoimmune type 1 diabetes (T1D) or polygenic type 2 diabetes (T2D), and many individuals remain unrecognized, incorrectly classified and thus inappropriately treated during their lifetime (5).

Much of the research on MODY has been conducted in European and East Asian populations (6–9). The MODY probability calculator developed by researchers at the University of Exeter (10) tends to overestimate the likelihood of MODY in non-White populations (11–13) who have high prevalence of young-onset T2D with polygenic background (14). Given this confounding factor, the best strategy to identify the “actionable subtypes” of MODY in racially and ethnically diverse population remains unknown. Whole-genome sequencing (WGS) is the gold standard to detect rare variants. In resource-constraint setting, screening for variants in MODY genes through targeted next-generation sequencing (NGS) in clinically-suspected individuals with familial, syndromic or atypical diabetes is a cost-effective approach to diagnose MODY (15).

*Glucokinase (GCK)*-MODY or MODY type 2 (MODY2) and *Hepatic Nuclear Factor 1 Alpha (HNF1A)* -MODY or MODY type 3 (MODY3) account for the majority of confirmed MODY cases (6–9). Individuals with *HNF1A*-MODY, especially if diagnosed early with preserved beta-cell function, respond well to low-cost sulfonylurea (SU) and dipeptidyl peptidase 4 inhibitor (DPP4i) without the need of insulin (16). There is also emerging evidence suggesting that patients with *GCK*-MODY may respond to GK activator (GKA) with restored glucose sensitivity and insulin secretion as well as increased time in range for glucose during continuous glucose monitoring (17). However, diagnostic yields of genetic testing for MODY vary across studies and are influenced by factors such as ethnicity, clinical criteria and sequencing coverage.

Cohort analysis on molecular testing in consecutive individuals with young-onset diabetes (YOD) diagnosed before age of 40 in Hong Kong or clinically-suspected MODY in India indicated lower detection rates than in European cohorts (8, 11, 13). Diagnostic challenges of MODY in Asian populations are compounded by the young age-at-diagnosis, lean body mass index (BMI) and strong family history (14). We have previously used targeted sequencing to diagnose MODY in Thai individuals with atypical diabetes (18, 19). Other Thai studies had identified MODY cases in children negative for autoimmune antibodies (20–22). This study aimed to evaluate the diagnostic yield and clinical impact of targeted gene panel testing in real-world setting including Thai individuals attending a specialized diabetes outpatient clinic.

## Methods

We conducted a retrospective analysis on all probands with atypical diabetes who attended Vimut-Theptarin Hospital (formerly Theptarin Hospital), Bangkok, Thailand and underwent genetic testing panel for monogenic diabetes between 2021 and 2025. Genetic testing with out-of-pocket payment was recommended at the discretion of the attending diabetologists based on their clinical evaluation. In this specialist care setting, we adopted a low threshold for recommending genetic testing in individuals with atypical features of diabetes. These included age at diagnosis before 40 years, BMI <25 kg/m^2^, random plasma C-peptide levels ≥ 0.1 ng/mL after at least three years of clinically-diagnosed T1D, syndromic features such as organ abnormalities or non-classical T1D or T2D presentations. All patients were negative for anti-glutamic acid decarboxylase antibodies (anti-GAD) antibodies. Anti-GAD was assessed by ELISA method (RSR ^®^, UK). Cut-off point for anti-GAD positivity is 5 U/mL with a specificity of 98% and sensitivity of 92%. Exclusion criteria included incomplete medical records, non-Thai nationality, or age <15 years at the time of genetic testing. Clinical data collected included sex, age-at-diabetes-diagnosis, presenting features, family history, physical examination findings, prior anti-diabetic medications, and age at genetic testing. We reported the genetic variants and variant classification as retrieved from formal laboratory reports. The study was approved by the Ethics Committees of the Vimut-Theptarin Hospital (EC No.1/2024).

Blood samples were sequenced for a panel of 33 genes relevant to monogenic diabetes using the NGS platform (Illumina, San Diego, CA, USA) and a mitochondrial mutation for maternally-inherited deafness and diabetes (MIDD, mt A3243G) (GemVCare, Shatin, Hong Kong) (8). These genes included *ABCC8*, *AKT2*, *APPL1*, *CEL*, *CISD2*, *DCAF17*, *DNAJC3*, *DYRK1B*, *GATA4*, *GATA6*, *GCK*, *HNF1A*, *HNF1B*, *HNF4A*, *INS*, *INSR*, *KCNJ11*, *LMNA*, *NEUROD1*, *PAX6, PCBD1*, *PDX1*, *PIK3R1*, *PLIN1*, *POLD1*, *PPARG*, *PPP1R15B*, *RFX6*, *SLC29A3*, *TRMT10A*, *WFS1, ZBTB20*, and *ZFP57*. The sequencing regions covered exons and flanking regions located within 25 base pairs (bp) upstream and downstream of each exon. Due to technical limitations in primer design, *CEL* (87.3% of bp), *GATA4* (95.2% of bp), *KCNJ11* (90.1% of bp), *NEUROD1* (96.3% of bp), *PDX1* (94.0% of bp), and *POLD1* (96.4% of bp) were only partially sequenced within the entire of each gene. Multiplex ligation-dependent probe amplification (MLPA) was used routinely to detect heterozygous deletions or duplications in *GCK, HNF1A, HNF1B, HNF4A* genes. Variant classification included “pathogenic” (P), “likely pathogenic” (LP), “variants of uncertain significance” (VUS), “likely benign” and “benign” based on combined scored evidence criteria in accordance to American College of Medical Genetics and Genomics (ACMG) and the Association for Molecular Pathology (AMP) Guidelines (23). Patients were considered to have MODY if they were heterozygous or homozygous carriers of P/LP variants in genes with autosomal dominant inheritance, or homozygous or compound heterozygous carriers of P/LP variants in genes with autosomal recessive inheritance. Copy number variations (CNV) in the *hepatocyte nuclear factor 1 beta* gene (*HNF1B*) region were analyzed by using an open‐source CNV detection algorithm CNVPanelizer (Bioconductor) and validated by multiplex ligation‐dependent probe assay (MLPA) (SALSA MLPA P241 MODY kit, MRC Holland, Amsterdam, Netherlands). Segregation analysis was performed on the family members of the proband, wherever available, to identify if the variant was inherited or *de novo*.

### Statistical analysis

Statistical analyses were performed using the Statistical Package for the Social Sciences (SPSS) version 24.0 (IBM Corp., Armonk, NY, USA). Continuous variables with normal distribution were expressed as mean ± standard deviation (SD), while categorical variables were presented as proportions. The Kolmogorov-Smirnov test was used to test the normality of the dataset. Clinical characteristics and laboratory data were compared between individuals with or without a confirmed MODY diagnosis. Between-group comparisons were performed using Student’s *t‐test* for continuous variables, chi‐squared test or Fisher’s Exact test for categorical variables and Wilcoxon rank‐sum test for non-normally distributed data. A two-sided *P-value* of <0.05 was considered significant.

## Results

Between 2019 and 2025, 33 probands with atypical diabetes underwent targeted genetic panel testing for monogenic diabetes using NGS and MLPA as appropriate. According to our diabetes registry data, during the study period, approximately 200–300 new cases attended our diabetes center. We estimated that 5% of them had features of atypical diabetes. Therefore, at least 105 cases during the study period had been offered genetic testing. At the end, 33 cases underwent genetic testing (31.4%). The mean age-at-diabetes-diagnosis was 34.4 ± 14.4 years, mean BMI was 23.7 ± 3.3 kg/m², and 39.3% were treated with insulin. The mean age at genetic testing was 39.8 ± 12.1 years with a mean of 5.4 years from first diagnosis of diabetes. None of the individuals reported parental consanguinity.

**Figure 1:**
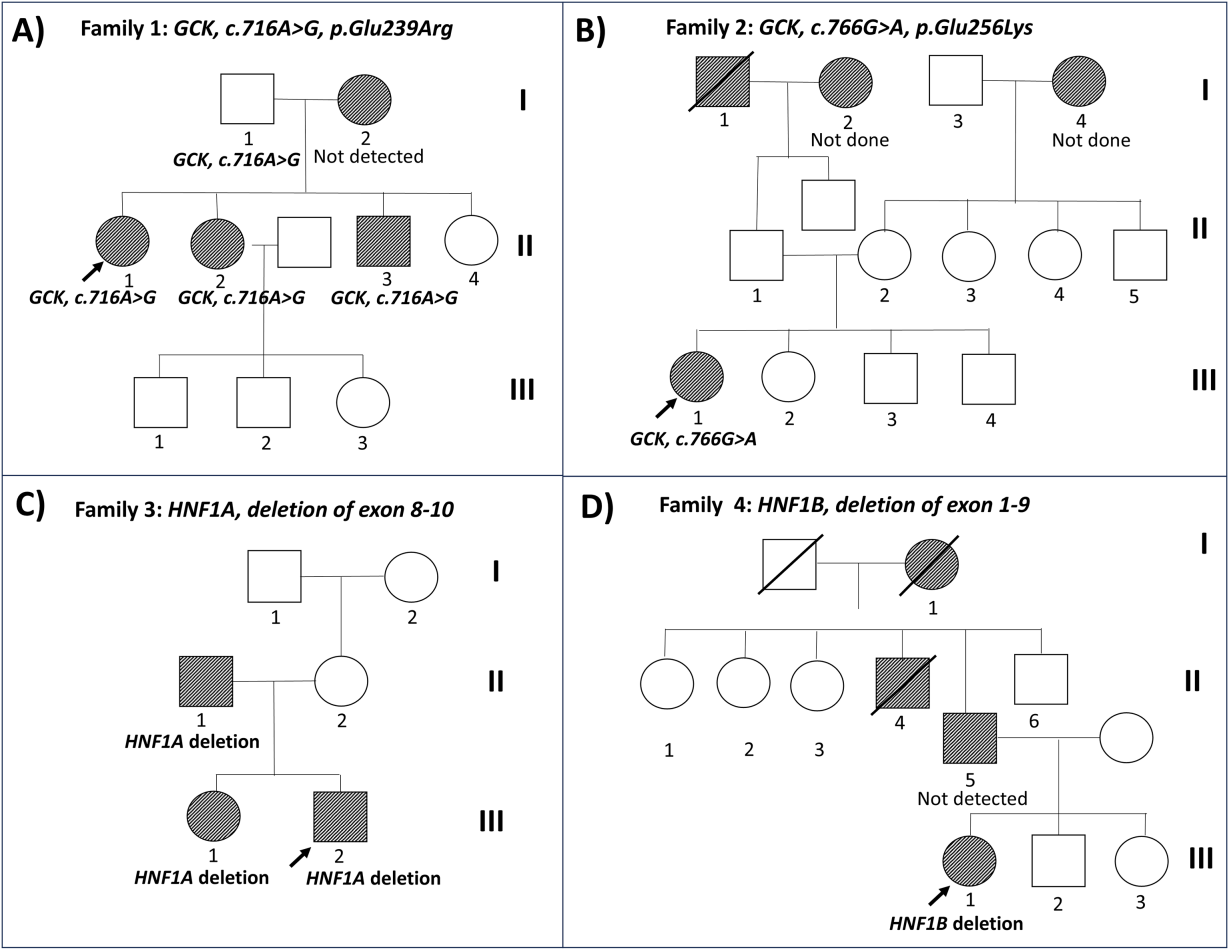
Pedigree of 4 Thai probands with molecularly-confirmed maturity-onset diabetes of the young (MODY) amongst 33 probands recruited in 2019–2025 **(A)** Family with *GCK*-MODY (c.716A>G) **(B)** Family with *GCK*-MODY (c.766G>A) **(C)** Family with *HNF1A*-MODY (deletion of exon 8-10) **(D)** Family with *HNF1B*-MODY (deletion of exon 1-9). An arrow indicates the proband. Filled symbols indicate diabetes and non-filled symbols indicate normal or unknown glucose tolerance.

**Table 1** compared individuals with or without a confirmed MODY diagnosis with the MODY patients having lower A1C (6.0 ± 0.8% vs 9.8 ± 2.6%, *P=*0.020) than those tested negative. A P/LP variant was identified in 4 of 33 probands (12.1%). The most frequently identified variants were located in *GCK* (n=2; 50.0%), followed by *HNF1A* (n=1; 25.0%), and *HNF1B* (n=1; 25.0%). The diagnoses of MODY led to changes in clinical management and facilitated cascade testing with identification of additional MODY cases among family members (**Figure 1**). Most of the relatives of probands with *GCK-*MODY and *HNF1A*-MODY had concomitant obesity-related insulin resistance.

**Table 1:** Demographic and clinical characteristics of all patients with atypical diabetes who underwent targeted next-generation sequencing.

Clinical parameters	All patients (N = 33)	Confirmed MODY with P/LP variants (N = 4, 12.1%)	P/LP variants of MODY genes not detected (N = 29, 87.9%)	*P-value*
Age at genetic testing (years)	39.8 ± 12.1	39.8 ± 15.1	40.4 ± 12.6	0.438
Age at diagnosis of diabetes (years)	34.4 ± 14.4	34.9 ± 6.6	33.7 ± 14.0	0.403
% Female	60.6%	75.0%	58.6%	0.481
BMI at diagnosis of diagnosis (kg/m^2^)	23.7 ± 3.3	21.1 ± 3.4	24.1 ± 3.1	0.089
History of DKA (%)	12.1%	0%	13.8%	0.321
A1C at diagnosis of diabetes (%)	9.3 ± 2.8	6.0 ± 0.8	9.8 ± 2.6	0.020
A1C at genetic testing (%)^*^	6.8 (5.9,8.9)	6.4 (5.4,7.8)	6.9 (5.9,9.5)	0.424
Treatments (%) • Diet control • Metformin only • Combined oral glucose lowering drugs • Insulin	15.2% 15.2% 30.3% 39.3%	75.0% 0% 25.0% 0%	6.9% 17.2% 31.0% 44.9%	0.007

^*^Data were presented as median (interquartile range) and Wilcoxon rank‐sum test was used for non-normally distributed data. P = Pathogenic variant/ LP = Likely Pathogenic variant. Bold values mean P-value significantly at less than 0.05.

### *GCK*−MODY

Two probands were identified with *GCK*-MODY during the study period. The first proband was a 41-year-old non-obese female, diagnosed with T2D at the age of 37, with a fasting plasma glucose (FPG) of 150 mg/dL and glycated hemoglobin (A1C) of 7.0%. She was initiated on metformin 1000 mg daily with an A1C range of 5.9-7.1%. Following the diagnosis of a paternally-inherited *GCK*-MODY due to a pathogenic missense variant (c.716 A>G) in the *GCK* gene (**Figure 1A**), metformin was discontinued. Cascade genetic testing revealed that her younger sister and brother carried the same variant. Her younger sister had known history of T2D treated with multiple oral anti-diabetic agents with a A1C range of 7.5%-8.3%. She also had hypertension, obesity (BMI 28.6 kg/m²) and proteinuria. Her brother who was also a carrier of the variant had diabetes upon screening.

The second proband was a 26-year-old female with a lean BMI of 16.7 kg/m^2^. She was diagnosed with diabetes during a routine medical check-up with FPG 132 mg/dL and A1C 6.6%. Although her parents did not have diabetes, both maternal and paternal grandparents had a history of diabetes (**Figure 1B**). A pathogenic missense variant (c.766 G>A) in the *GCK* gene was identified and the patient continued to be managed by lifestyle modification according to current recommendation that *GCK*-MODY does not require treatment.

### *HNF1A*−MODY

One proband with *HNF1A* pathogenic variant was identified in our case series. A 34-year-old, non-obese Thai man was diagnosed with T2D at the age of 30 treated with oral anti-diabetic medications. He presented to our clinic four years later due to poor glycemic control. Detailed history taking did not reveal history of ketosis and he was considered to having lean T2D (BMI 23.8 kg/m^2^) with a FPG of 130 mg/dL, and A1C of 7.8%. He was started on metformin 1,000 mg daily with a A1C range of 6.7-8.1% during follow-up. His A1C fluctuated between 7.5% and 8.8% during the next 12 months and required additional anti-diabetic medications with a latest A1C of 8.2%. He had microalbuminuria but no evidence of retinopathy. Both his father and older sister had diagnosis of T2D (**Figure 1C**).

A paternally-inherited heterozygous deletion of *HNF1A* exon 8–10 was identified, confirming a diagnosis of *HNF1A*-MODY in the index case. Following the genetic diagnosis, metformin and sitagliptin were discontinued. Over a 6-month period, glycemic control improved with SU monotherapy using oral gliclazide 60 mg once daily. His A1C decreased from 8.2% to 7.1% within this period. Subsequent cascade genetic testing revealed that his older sister with YOD also carried the same *HNF1A* variant. She was noted to have morbid obesity with a BMI of 38.6 kg/m².

### *HNF1B*−MODY

One proband with a pathogenic *HNF1B* variant was identified in this case series. She was a 35-year-old, non-obese woman with YOD and negative for autoantibodies. She returned to our diabetes clinic after having defaulted for 2 years. At the age of 30, she was diagnosed with T2D based on 75-g oral glucose tolerance test (OGTT) during an annual health check-up prompted by elevated FPG. At that time, she was asymptomatic with a BMI of 21.4 kg/m^2^; her post-load PG was 219 mg/dL and A1C was 6.3%. She was started on metformin 1,000 mg/day and gemfibrozil 300 mg/day, with regular follow-up for two years. She then got pregnant and all medications were stopped. When she returned to our clinic at the age of 35, she remained well and was not on any medication. Detailed history taking revealed normal childhood development. At the age of 12, she was investigated for urinary tract infection and diagnosed to have atrophic left kidney on ultrasonography. Her renal function remained normal. Her father was diagnosed to have T2D at the age of 39 during medical check-up (**Figure 1D**). He was overweight (BMI 25.6 kg/m^2^) with hypertension and hypercholesterolemia.

Given the patient’s young age-at-diagnosis, left renal atrophy, and paternal history of diabetes, the possibility of MODY was suspected. Genetic panel revealed negative results but heterozygous deletion of exons 1–9 of *HNF1B* gene was detected using MLPA. Further MLPA testing for *HNF1B* in her parents, her younger siblings, and her son showed no deletion of *HNF1B*, suggesting *de novo* deletion of *HNF1B* in the index patient. Low-pass WGS confirmed *HNF1B* deletion as part of the 17q12 deletion syndrome. Computed tomography of the abdomen and pelvis showed hypoplasia of the body and tail of the pancreas, and atrophic dysplastic left kidney. No abnormalities of genitourinary system were found. Five years after the diagnosis of *HNF1B*-MODY, the patient maintained good glycemic control on multiple oral anti-diabetic medications with normal renal function.

## Discussion

In this case series, we identified MODY variants in 12.1% of Thai patients with atypical diabetes attending a specialist diabetes clinic. Amongst the 4 positive cases, 2 had *GCK*-MODY, 1 had *HNF1A*-MODY and 1 had *HNF1B*-MODY. These diagnoses had led to treatment changes with additional cases identified through cascade screening. Several of the family members were found to have obesity-associated insulin resistance contributing to their clinical presentation. To our knowledge, this is the first study to evaluate the impact of targeted gene sequencing to detect MODY in Thai patients with atypical diabetes in a non-research setting.

The frequency of MODY in non-European MODY case series or cohorts varied from 1.3 to 21.1% (8, 9, 11, 13, 24–32) depending on selection criteria. With more stringent criteria, the detection rate (12.1%) in our case series was similar to that reported in Indian (15.5%) (11), Singaporean (15.5%) (25), and Taiwanese (13.8%) (31) cohorts as shown in **Table 2**. In research setting, the prevalence of MODY was 1.3-1.9% in consecutive cohorts of Chinese patients with YOD with less strict inclusion criteria (8, 13). These discrepancies highlight the importance of selection criteria to increase the yield for genetic testing in real-world practice.

**Table 2:** Summary of diagnostic yield for molecularly-confirmed maturity-onset diabetes of the young (MODY) in Asian cohorts diagnosed with adult-onset type 2 diabetes.

Country	Setting	Sequencing method	Diagnostic yield	Distribution of MODY genes
China (Ref. No 9)	Research setting Non‐type 1 diabetes diagnosed at age ≤ 40 years during 2017-2023 Mean age-at-diagnosis 24.3 years	Whole Exome Sequencing	37/202 (18.3%)	GCK (n=5) HNF1A (n=3) HNF4A (n=3) HNF1B (n=3) Others (n=23)
Hong Kong^*^ (Ref. No 8) (Ref. No 13)	Research setting Non‐type 1 diabetes diagnosed at age ≤ 40 years during 1995-2012 Mean age-at-diagnosis 33.0 years Research setting Non‐type 1 diabetes diagnosed at age ≤ 40 years during 2020-2021 Mean age-at-diagnosis 34.0 years	33 monogenic diabetes panel by NGS 34 monogenic diabetes panel by NGS	19/1021 (1.9%) 11/822 (1.3%)	GCK (n=6) HNF1A (n=9) HNF4A (n=1) HNF1B (n=3) GCK (n=4) HNF1A (n=6) HNF4A (n=0) HNF1B (n=1)
India (Ref. No 11)	Research setting Non‐type 1 diabetes diagnosed at age ≤ 30 years during 2020-2024 Mean age-at-diagnosis 19.8 years	75 monogenic diabetes panel by NGS	120/774 (15.5%)	GCK (n=4) HNF1A (n=39) HNF4A (n=20) HNF1B (n=16) Others (n=41)
Japan (Ref. No 30) (Ref. No 32)	Research setting Non‐type 1 diabetes diagnosed at age <50 years during 2017-2020 Mean age-at-diagnosis 31.0 years Research setting Non‐type 1 diabetes diagnosed at age <35 years during 2019-2024, nationwide Median age-at-diagnosis 15.0 years	34 monogenic diabetes panel by NGS 11 monogenic diabetes panel by NGS	5/56 (8.9%) 67/232 (28.9%)	GCK (n=2) HNF1A (n=0) HNF4A (n=2) HNF1B (n=0) Others (n=1) GCK (n=25) HNF1A (n=22) HNF4A (n=6) HNF1B (n=7) Others (n=7)
Korea (Ref. No 29)	Research setting Non‐type 1 diabetes diagnosed at age ≤ 30 years during 2001-2018 Mean age-at-diagnosis 20.2 years	30 monogenic diabetes panel by NGS	23/109 (21.1%)	GCK (n=7) HNF1A (n=3) HNF4A (n=3) HNF1B (n=1) Others (n=9)
Singapore (Ref. No 25)	Research setting Non‐type 1 diabetes diagnosed at age ≤ 45 years during 2013-2015 Mean age-at- diagnosis 25.0 years	75 monogenic diabetes panel by NGS	13/84 (15.5%)	GCK (n=1) HNF1A (n=2) HNF4A (n=1) HNF1B (n=0) Others (n=9)
Taiwan (Ref. No 31)	Routine clinical setting Non‐type 1 diabetes diagnosed at age ≤ 30 years Mean age-at-diagnosis Not reported	Whole Exome Sequencing	11/80 (13.8%)	GCK (n=5) HNF1A (n=0) HNF4A (n=1) HNF1B (n=2) Others (n=3)
Thailand (Ref. No 20)	Research setting Non‐type 1 diabetes diagnosed at age ≤ 35 years in 2007 Mean age-at-diagnosis 16.0 years Routine clinical setting Present study Atypical diabetes from a private specialist diabetes center during 2019-2025 Mean age-at-diagnosis 34.4 years	Sequencing of six known genes responsible for MODY (HNF4A , GCK, HNF1A, IPF-1, HNF-1B, NeuroD1) 33 monogenic diabetes panel by NGS	6/51 (11.8%) 4/33 (12.1%)	GCK (n=1) HNF1A (n=2) HNF4A (n=1) HNF1B (n=0) Others (n=2) GCK (n=2) HNF1A (n=1) HNF4A (n=0) HNF1B (n=1)

*Both Hong Kong cohorts were studied in consecutive cases without any selection criteria except for young age-at-diagnosis < 40 years.

Three decades of research has advanced our understanding of the causes and management of monogenic diabetes. However, there remain significant care gaps in diagnosing individuals with MODY, particularly among Asian populations (8). The MODY probability calculator developed in Europeans which included clinical and laboratory-based predictors overestimated the probability of MODY in non-Europeans (12, 13, 28). The optimal cutoff of these scores needs to balance sensitivity and specificity which are influenced by the prevalence of MODY in a specific population. Moreover, the candidate list of genes for monogenic diabetes has expanded since the development of the first MODY probability calculator based on Europeans with an age range of 1–35 years with confirmed diagnosis of *HNF1A*, *HNF4A* or *GCK*-MODY (10).

Accurate identification of Asian patients with MODY is challenging given the high background prevalence of YOD due to polygenic causes mainly implicated in beta-cell dysfunction (14). The high prevalence of VUS in Asians with under-representation in global variant databases, calls for more family-based studies to ascertain their significance. At our center, diabetologists have low threshold to order genetic testing for MODY in patients with atypical T1D or T2D, especially in those with YOD, lean BMI and residual beta-cell function. Unidentified MODY-related genes in patients with atypical diabetes might be due to causal variants not detected by conventional targeted exome sequencing e.g. variants in non-coding and regulatory regions. Patients with variants of unknown significance (VUS) might be novel variants but were not considered for reporting based on conventional criteria. Despite strong clinical suspicion, the significance of these VUS need further evaluation based on functional analysis or co-segregation amongst affected family members. Given the gap in genomic knowledge in non-European population, our data highlighted the heterogeneity of atypical diabetes in Asians calling for more family-based and register-based cohorts accompanied by biobanks, use of advanced technology and collaborative analysis to improve the precision of diagnosis and treatment of atypical diabetes in non-European populations.

From a clinical perspective, a genetic diagnosis of MODY will facilitate cascade screening to detect new cases of MODY or reclassify diabetes to MODY in family members. Of note, many of our patients with MODY also had obesity as a typical feature of T2D. This hybrid form of T2D and MODY is increasingly recognized across various MODY subtypes (18, 33–35). Classical teaching states that typical *GCK*-MODY would not require pharmacologic treatment due to its mild and non-progressive nature (36). However, since *GCK*-MODY, polygenic-T2D and obesity-associated insulin resistance are not mutually exclusive, these patients should benefit from conventional anti-diabetic medications to achieve glycemic control early as well as control of cardiovascular-kidney-metabolic risk factors for long-term organ protection. Besides, genetic analysis and Mendelian randomization analysis suggested that *GCK* might play causal role in cardiovascular-kidney complications (37).

For individuals with *HNF1A*-MODY, SU is considered the first-line therapy. Nevertheless, SU may increase the risk of hypoglycemia and may not provide sustained glycemic control over time. Recent studies have demonstrated the benefits of incretin-based therapies and sodium-glucose cotransporter-2 inhibitor (SGLT2i) in individuals with *HNF1A*-MODY (16, 38). Given the effects of glucotoxicity on beta-cell function, early control of hyperglycemia with low risk of hypoglycemia using any anti-diabetic medications can theoretically break the vicious cycle with sustained glycemic control and long-term benefits. Comparative targeted trials between SU or DPP4i or GKA versus other anti-diabetic medications will be informative in advancing our understanding and implementation of precision medicine in MODY subtypes.

*HNF1B*-MODY (formerly referred to as MODY5 or renal cysts and diabetes syndrome) accounts for 2-5% of all MODY subtypes (3). As previously reported by our group (19), the presence of CKD and/or genitourinary tract malformations should raise clinical suspicion for *HNF1B*-MODY, particularly in patients with young-onset CKD with renal abnormalities with or without pancreatic hypoplasia or aplasia (39). Our case also highlights the utility of MLPA in detecting large genomic rearrangements such as deletions or insertions, which may not be captured by standard NGS panels. The presence of a deletion in *HNF1B* should prompt consideration of 17q12 deletion syndrome (40) as confirmed by low-pass WGS. This syndrome is characterized by multi-system manifestations. Early diagnosis and cascade screening might help prevent progressive beta-cell and kidney dysfunction. Our patient with *HNF1B*-MODY harbored a *de novo* deletion, a finding reported in up to 50% of affected individuals (41). Thus, the recognition of syndromic features, such as atypical organ or developmental abnormalities, regardless of family history, should alert the physician to consider offering genetic testing for MODY genes (42, 43).

Our study has several limitations. Due to the setting of a private specialist diabetes center and high cost of genetic testing, we might have selected patients with greater financial means to undergo genetic testing. Referral for genetic testing was based on clinical judgment of the attending diabetologists, without standardized inclusion and exclusion criteria. Increased clinical experience with MODY patients and increased affordability of the genetic tests may contribute to temporal variability in diagnostic yield. During this study period, we estimated that 2-5% of our clinic attendees had features of atypical diabetes.

At our center, genetic testing was initially offered mainly to individuals with diabetes affecting at least three generations. With more experience, we gradually broadened the referral criteria including patients with atypical and syndromic features. In some patients, missing data such as C-peptide during follow up did not allow evaluation of progression of beta-cell function. We measured plasma C-peptide only in patients with atypical clinical features and patients with suspected T1D. Moreover, due to the limited coverage of the gene panel, novel or unidentified MODY genes might have been missed (44). As WES and WGS become increasingly accessible and affordable, more novel MODY genes, supported by functional analysis or co-segregation studies amongst family members might be discovered in Thai population.

In conclusion, genetic testing for MODY in a private specialist diabetes center setting identified 12.1% of Thai patients with atypical diabetes harboring P/LP variants of MODY genes. Despite this relatively low yield, a confirmed diagnosis of MODY had led to meaningful actions including management strategies and cascade screening. The presence of obesity, lack of family history or history of diabetic ketoacidosis in some of these patients did not preclude the diagnosis of MODY. These findings support the multicausality of T2D where common and rare genetic variants may interact with other risk factors, notably obesity, to contribute to varying clinical presentations. This complexity calls for establishment of registers of atypical diabetes with systematic documentation of clinical and laboratory assessment including sequencing to improve the precision of diagnosis, prognosis, therapies and outcomes in these patients and their family members.

## Data Availability

The raw data supporting the conclusions of this article will be made available by the authors, without undue reservation.
